# Quantifying Molecular Disorder in Tri-Isopropyl Silane (TIPS) Pentacene Using Variable Coherence Transmission Electron Microscopy

**DOI:** 10.1021/acs.jpclett.3c01344

**Published:** 2023-09-06

**Authors:** F. Alanazi, A. S. Eggeman, K. Stavrou, A. Danos, A. P. Monkman, B. G. Mendis

**Affiliations:** † Department of Physics, Durham University, South Road, Durham DH1 3LE, U.K.; ‡ Department of Materials, University of Manchester, Oxford Road, Manchester M13 9PL, U.K.

## Abstract

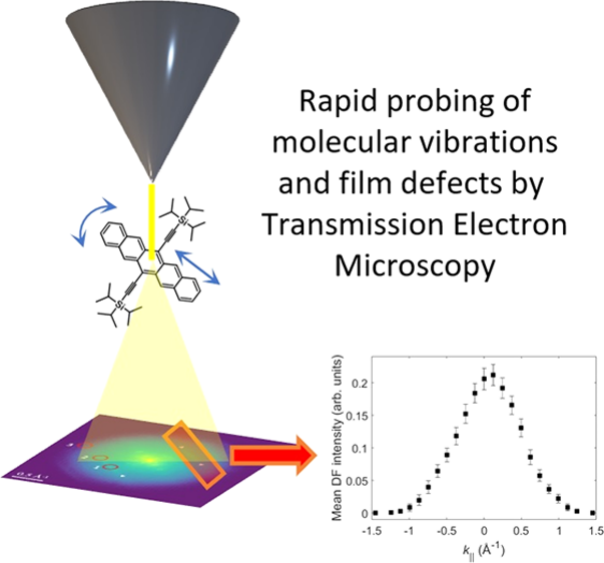

Structural disorder in molecular crystals is a fundamental limitation for achieving high charge carrier mobilities. Quantifying and uncovering the mechanistic origins of disorder are, however, extremely challenging. Here we use variable coherence transmission electron microscopy to analyze disorder in tri-isopropyl silane pentacene films, utilizing diffuse scattering that is present both as linear streaks and as a slowly varying, isotropic background. The former is due to thermal vibration of the pentacene molecules along their long axis, while the latter is due to static defects kinetically frozen during film deposition. The thermal vibrational amplitude is ∼0.4 Å, while the static displacement parameter in our simplified analysis is much larger (1.0 Å), because it represents the cumulative scattering of all defect configurations that are frozen in the film. Thin film fabrication therefore has an important effect on crystallinity; our technique can be readily used to compare samples prepared under different conditions.

Crystallinity plays a crucial role in the charge carrier mobility of polymer and small molecular organic thin films.^1,2^ Long range order promotes electron delocalization and band-like transport with high mobilities, while structural disorder creates localized traps and a less efficient hopping transport mechanism. Even in highly crystalline organic molecular semiconductors, such as tri-isopropyl silane (TIPS) pentacene or rubrene, there is evidence for dynamic disorder, which arises from vibrations of the molecular fragments about their mean lattice position.^3,4^ These vibrations are thermally driven and can have amplitudes as large as 0.5 Å,^3^ due to the relatively weak intermolecular van der Waals forces. This dramatically alters the orbital overlap between neighboring molecules and corresponding charge transfer integrals,^5^ so that as a general rule, electrons become more localized and hopping transport dominates. Changes in orbital overlap also strongly impact the ability of molecular pairs to undergo advanced photophysical processes such as singlet fission, for which TIPS pentacene has emerged as an exemplary system.^6^ In addition to thermal motion, structural defects can also be kinetically frozen into the thin film during processing. As an example, solvents with higher boiling points promote better crystallinity and therefore larger mobilities in spin-coated TIPS pentacene thin film transistors.^7^ These kinetically limited defects are described here as “static”, to distinguish them from the “dynamic” structural disorder caused by thermal vibrations.

Characterizing disorder at the molecular level is a highly complex and difficult challenge. Dynamic disorder gives rise to diffuse streaking in electron diffraction patterns.^3,4^ The electron diffraction pattern for a given molecular crystal structure can be simulated using the multislice method,^8^ and the intensity profile for the diffuse streak compared with experiment to establish its accuracy and estimate disorder. Multislice is however time-consuming for such large supercells, especially because many configurations of the dynamic disorder must be averaged to produce statistically representative results.^9^ An alternative method is fluctuation electron microscopy (FEM), which is a technique used to quantify medium range order in amorphous materials.^10−12^ FEM has been applied to amorphous inorganic semiconductors^10,13^ and bulk metallic glasses,^14,15^ although there are fewer examples of quantifying disorder in crystalline materials.^16^ Diffuse scattering has also been extensively investigated in protein crystallography, mainly using X-rays.^17−19^

Here we show that the “variable coherence” principle underpinning FEM can also be used to directly quantify structural disorder in TIPS pentacene without the need for complex computer simulations. In “variable coherence”, the coherence volume over which diffraction takes place is varied by suitably adjusting the detection parameters for electron scattering.^20^ For diffuse scattering to occur, the coherence volume must be larger than the characteristic length scale of the structural disorder; smaller coherence volumes will produce less diffuse intensity in the electron diffraction pattern. Thus, changes in the diffuse scattered intensity with the coherence volume can be used to extract structural information and the length scale of the disorder itself. The variable coherence method is demonstrated on experimental TIPS pentacene data, collected in four-dimensional (4D) scanning transmission electron microscopy (STEM) mode, where a focused electron beam is rastered over the specimen region of interest and a complete diffraction pattern acquired at each scan position.^21^ 4D STEM has had previous success characterizing beam sensitive materials, such as mapping π-stacking in conjugated polymers,^22^ as well as extracting the radial distribution function of amorphous organic polymer–small molecule blends.^23^ Strictly speaking, a 4D STEM setup is not essential, because the same analysis can be performed using selected area electron diffraction from a parallel electron beam. However, 4D STEM does provide the benefit of selecting specimen regions that are oriented away from strong diffraction conditions, an important prerequisite for the variable coherence method, which is based on kinematical scattering.

TIPS pentacene thin films were deposited on a glass substrate with a spin-coated PEDOT:PSS layer. The TIPS pentacene was purchased from Ossila Ltd. and had a purity of >99.9%, as stated by the manufacturer. It was dissolved in chlorobenzene at a concentration of 20 mg/mL and stirred overnight. The solution was then drop casted on top of the PEDOT:PSS film, in a glovebox under a nitrogen atmosphere, followed by postdeposition annealing at 100 °C for 5 min. For the preparation of the TEM specimen, the glass substrate was immersed in deionized water. After a few minutes, the PEDOT:PSS layer dissolved and the TIPS pentacene film floated to the surface. The film was then picked up with 250 mesh TEM copper grids.

4D STEM measurements were performed at the University of Manchester, using a 200 kV Thermo Fisher Scientific Talos field emission gun TEM instrument, equipped with a MerlinEM direct electron detector. The STEM probe semiconvergence angle was 0.6 mrad, and the first condenser lens excitation was set to its highest value (i.e. “spot size” 9) to improve electron beam coherence.^24^ The 4D STEM step size was 20 nm, and the pixel dwell time was 250 ms. The average number of electrons per scan position was 66, which gives a fluence of ∼0.03 electrons/Å^2^ for a 5 nm diffraction-limited probe diameter. These experimental conditions were found to limit electron beam damage of TIPS pentacene; i.e. there was no noticeable change in the electron diffraction pattern after a 4D STEM scan. The specimen thickness is 1.3–1.5 inelastic mean free paths (∼150 nm), although the exact thickness of the region analyzed is unknown. The film thickness was estimated from an electron energy loss spectrum (EELS)^25^ acquired in a Gatan Tridiem imaging filter and a 200 kV, JEOL 2100F FEG TEM instrument at Durham University. See the Supporting Information for the EELS spectrum.

Figure 1a shows a bright-field (BF) image of the TIPS pentacene film generated by positioning a virtual aperture over the unscattered beam in the 4D STEM data set. Bend contours from a [001] zone axis are visible across the entire field of view, indicating large, micrometer size crystalline grains. Note that the anomalous contrast for the first two pixel columns on the left-hand side is due to a STEM scan distortion. The diffraction pattern for the entire sample region, obtained by summing diffraction patterns at all STEM probe raster positions unaffected by scan distortion, is shown in Figure 1b. A logarithmic intensity scale is used to highlight weak features in the diffraction pattern. Apart from Bragg diffraction spots due to long range crystalline order, there is also an extensive diffuse background that appears as a circular halo centered around the unscattered beam. Superimposed on this background is further diffuse scattering in the form of linear streaks. There are two parallel sets of streaks, an inner streak indicated by arrows in Figure 1b and a weaker outer one. The latter lies just outside the circular diffuse background, which together with the differences in shape, suggests that the disorder for the two forms of diffuse scattering must have different origins.

**Figure 1:**
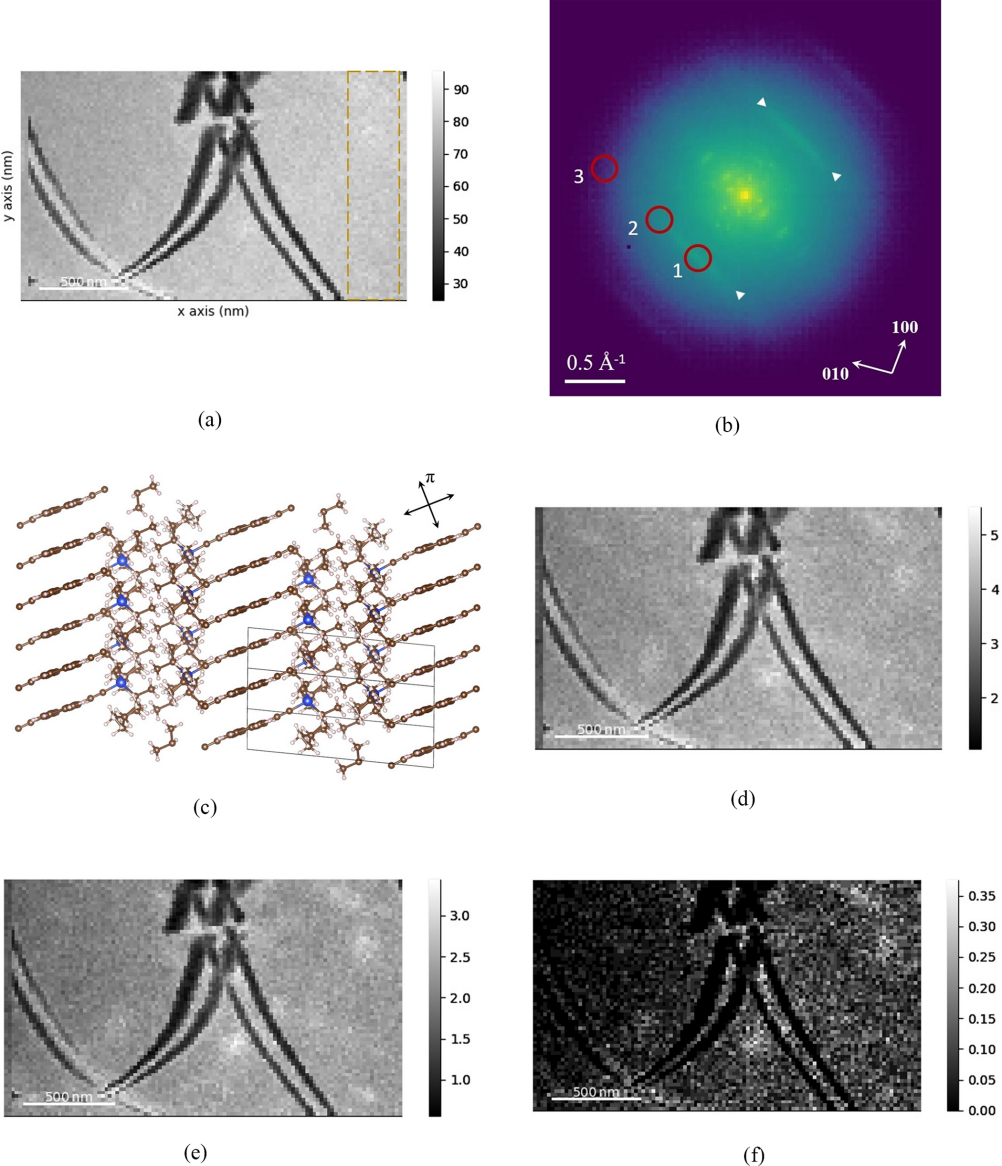
(a) Bright-field (BF) image of TIPS pentacene generated from a 4D STEM data set. The annotated box region on the right-hand side was chosen for further data analysis (Figure 3). The summed diffraction pattern (b) over the specimen area is displayed on a logarithmic intensity scale and shows diffuse streaking, indicated by arrows. (c) TIPS pentacene crystal structure viewed along the [210] direction. The unit cell boundaries are superimposed. Arrows indicate the directions of damped vibrations, including along the π-stacking direction. Dark-field (DF) images generated from a virtual aperture at positions 1–3 along the diffuse streak (Figure 1a) are shown in panels d–f, respectively. Note the change in intensity scale between the figures. The scale bar for the BF and DF images is 500 nm.

The diffuse streaking in TIPS pentacene has been discussed in detail previously^3^ and is caused by thermal vibrations of the pentacene molecules along their long axis, which is nearly parallel to [210]. To highlight this, Figure 1c shows a [210] projection of the triclinic TIPS pentacene crystal. Out-of-plane vibrations are suppressed by large force constants due to π-stacking, and in-plane vibrations perpendicular to the pentacene long axis are damped by enmeshing of the bulky TIPS substituents.^4^ Therefore, the only degree of freedom is along the pentacene long axis, i.e. in and out of the page in Figure 1c. The correlated motion, which is active during the room-temperature 4D STEM measurements, manifests as the diffuse streaks in the electron diffraction pattern. In contrast, the isotropic nature of the circular diffuse background suggests that this scattering is due to more random disorder. We reasonably propose that there is static disorder kinetically frozen into the drop cast thin film as it dries. The finite time for solidification means that some of the TIPS pentacene molecules may deviate from their ideal lattice positions and orientations. For a perfect crystal under parallel beam illumination, the Bragg diffraction peaks would be δ-functions with no intensity between them. Static disorder causes the Bragg diffraction peaks and unscattered beam to broaden out, with the long tails producing diffuse background intensity between the Bragg “spots”. This is the origin of the circular diffuse background.

Beyond qualitative identification, dynamic disorder and associated linear molecular displacements can also be quantified using variable coherence electron microscopy. This involves generating a series of dark-field (DF) images from virtual apertures positioned along the length of the diffuse streak. Three representative DF images are shown in Figure 1d–f, corresponding to virtual aperture positions labeled 1–3 in Figure 1b. As the virtual aperture is shifted from the center of the diffuse streak, the contrast of the bend contour and mean intensity of the DF image decrease. This is due to a shrinking coherence volume, which suppresses the ability to form diffraction contrast features such as bend contours.^20^ DF images acquired further along the diffuse streak are, therefore, more incoherent.

A kinematical scattering theory for the DF image intensity will now be presented, where individual molecules are modeled as point scattering objects. Following Treacy and Gibson, the direction of electron beam scattering by a molecule is reversed by invoking the principle of reciprocity.^20^ This is illustrated schematically in Figure 2a, which shows the wavevector **k** (effectively the scattered beam in reverse) scattered by molecule *j* along wavevector **k′** that falls within the STEM probe forming aperture. In the far field, the scattered wave function is *f*_m_(Δ**k**)exp(−2*πi*Δ**k**·**r**_*j*_), where *f*_m_(Δ**k**) is the scattering factor for the molecule at position vector **r**_*j*_ and Δ**k** = **k′** – **k** is the scattering vector. The realspace image wave function ψ(**r**) is obtained by an inverse Fourier transform:
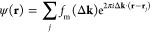
1The summation is over all molecules *j* within the illumination volume. For the sake of simplicity, we assume an infinitesimally small probe forming aperture, so that **k′** is parallel to the optic axis; consequently, there are no lens aberrations. This condition is approximately satisfied in our measurements, because the STEM probe semiconvergence angle is only 0.6 mrad. The real space image intensity *I* is the square modulus of eq 1, i.e.
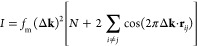
2where *N* is the number of molecules. The first term *Nf*_m_(Δ**k)**^2^ is the incoherent intensity, while the summation is the coherence volume and represents pairwise interference between molecules *i* and *j* separated by **r**_*ij*_ = **r**_*i*_ – **r***_j_*. Due to structural disorder, we assume interference is non-negligible only for nearest-neighbor molecules. This is because the cos(2πΔ**k**·**r**_*ij*_) term will be rapidly oscillating for molecular pairs that are farther apart, i.e. large **r**_*ij*_. Thermal vibration of TIPS pentacene displaces the molecules along their long axis, so that in the small displacement limit
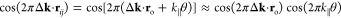
3where **r**_o_ is the equilibrium position vector between nearest-neighbor molecular pairs, θ is the change in nearest-neighbor spacing **r**_i*j*_ along the molecule long axis, and *k*_∥_ is the component of Δ**k** parallel to the diffuse streak, i.e. perpendicular to the displacement in real space. Assuming a Gaussian distribution of θ values with standard deviation σ, the mean diffuse scattered intensity *I*_diff_ is given by^26^
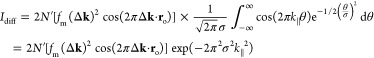
4where *N*′ is the number of nearest-neighbor molecule pairs. The logarithm of diffuse intensity *I*_diff_ varies linearly with *k*_∥_^2^, provided that the terms within the square brackets in eq 4 are approximately constant. This will be discussed in more detail below. The linear trend is due to the size of the coherence volume along the molecule long axis, which is given by exp(−2π^2^σ^2^*k*_∥_^2^) in eq 4. Note that the coherence volume in this instance is governed by an integration over molecular displacements θ rather than an integration over the detector plane. A similar effect has been observed with annular DF imaging,^20^ where suppression of the coherence volume along the electron optic axis is due to thermal vibration of the atoms. The gradient of a plot of ln(*I*_diff_) versus *k*_∥_^2^ is −2π^2^σ^2^, which yields the dynamic disorder parameter σ. Although the dynamic disorder is time-dependent, the swift passage of the STEM beam through a thin sample means that the molecules are effectively stationary during electron scattering.^9^ Therefore, σ must be interpreted as the standard deviation of the dynamic disorder at a fixed point in time.

**Figure 2:**
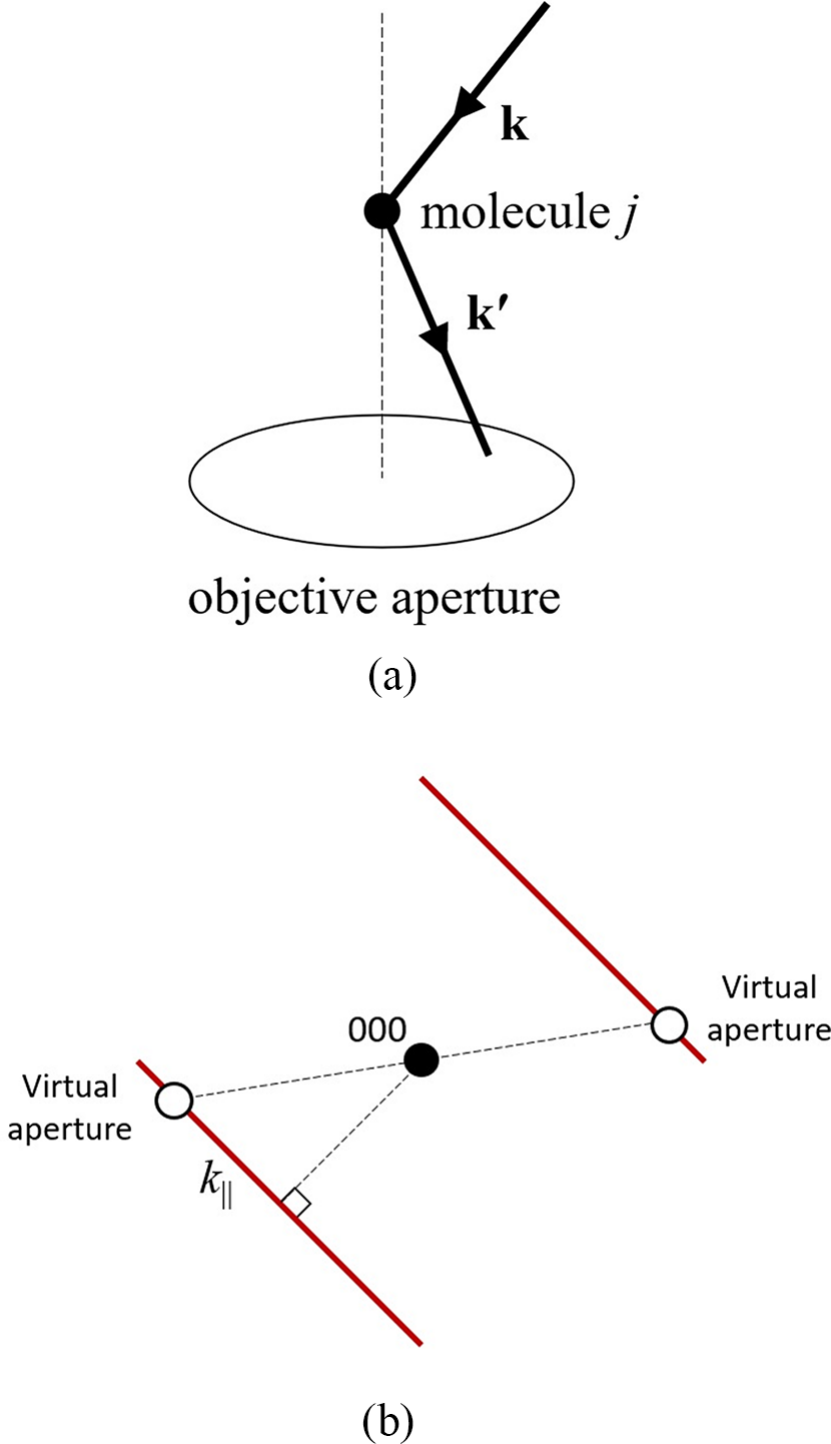
(a) Schematic depicting kinematical scattering of the electron beam by a molecule according to the principle of reciprocity. See the text for more details. (b) Schematic of the virtual apertures used to calculate the mean dark-field (DF) intensity for the diffuse streak (red lines). 000 represents the unscattered beam.

To test this model, a series of DF images were generated by positioning a virtual aperture at different wavenumbers *k*_∥_ along the diffuse streak. A Friedel pair of 3 × 3 pixel virtual apertures was used for improved statistics (Figure 2b; the pixel size in reciprocal space is 0.03 Å^–1^). Furthermore, only specimen regions that did not satisfy strong diffraction conditions were analyzed, because Bragg diffraction is not included in our kinematical model and can therefore result in artifacts. For example, the DF images in Figure 1d–f have the same bend contour contrast as the BF image (Figure 1a). This is because the unscattered beam intensity is depleted at the bend contour due to strong Bragg diffraction, so that the intensity available for diffuse scattering along the streak direction is lower. The annotated region on the right-hand side of Figure 1a was selected for analysis, away from the bend contour, and had a total of 990 scan positions. The mean DF intensity for this specimen region was calculated, and its profile as a function of *k*_∥_ is shown in Figure 3a. The peak maximum is slightly shifted (by one data point) to positive *k*_∥_ values, which is likely due to an error in estimating the true origin of the diffuse streak.

**Figure 3:**
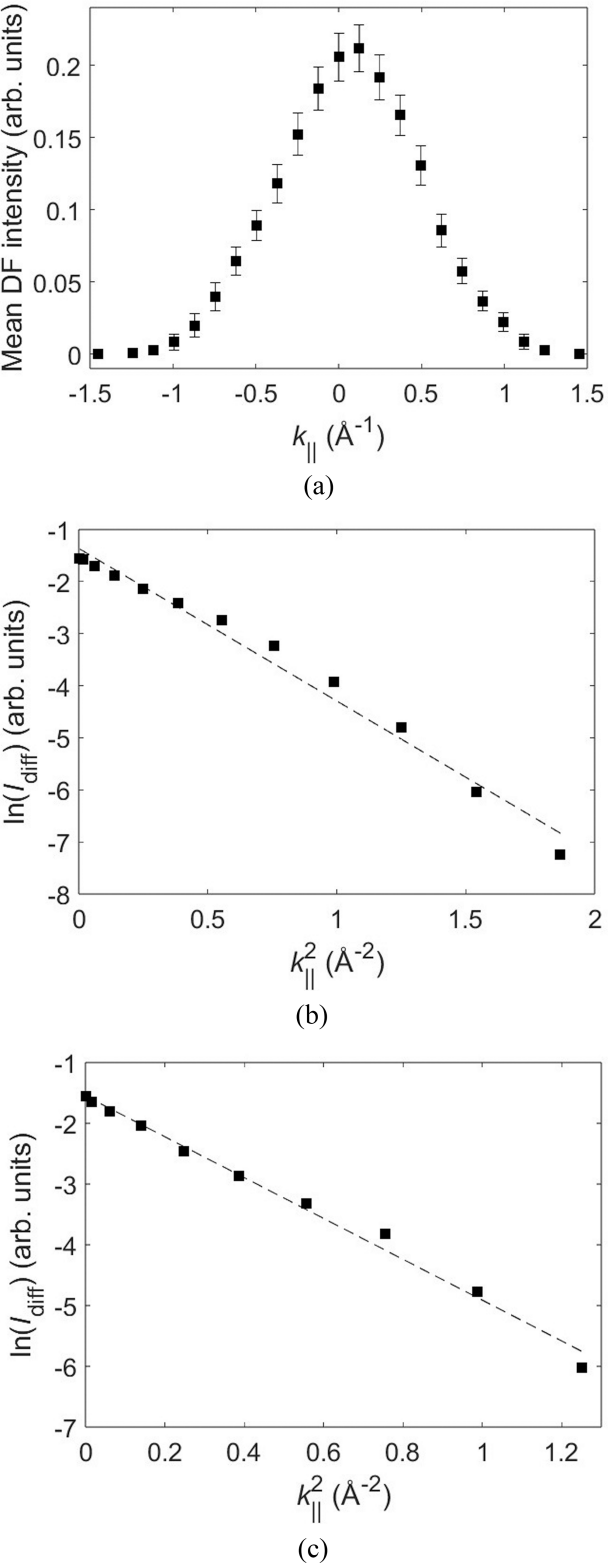
(a) Mean dark-field (DF) intensity plotted as a function of wavenumber *k*_∥_ along the diffuse streak. Plots of ln(*I*_diff_) vs *k*_∥_^2^ for the left- and right-hand portions of the intensity profile are shown in panels b and c, respectively. The equations for the best fit straight lines in panels b and c are *y* = −2.9291*x* – 1.3645 and *y* = −3.3613*x* – 1.5505, respectively.

Panels b and c of Figure 3 show graphs of ln(*I*_diff_) versus *k*_∥_^2^ generated from the left- and right-hand side portions of the intensity profile in Figure 3a. The origin of *k*_∥_ has been corrected to coincide with the peak of the mean DF intensity (this, however, did not have a significant effect on the final result). From eq 2, the DF signal contains an incoherent background intensity component. Furthermore, the diffuse streak is superimposed on a slowly varying static disorder background intensity. Both of these contributions must be subtracted from the mean DF intensity to obtain theintensity *I*_diff_ arising only from dynamic disorder. The background was taken to be the mean DF intensity at the largest *k*_∥_ wavenumbers, i.e. the extreme left and extreme right data points in Figure 3a. The resulting graphs (Figure 3b,c) showed a good linear fit across the entire range of *k*_∥_ values, with standard deviation σ for dynamic disorder estimated to be 0.38 ± 0.03 and 0.41 ± 0.05 Å, respectively. In comparison, a smaller σ value of 0.13 Å is obtained by molecular dynamics and multislice simulation of electron diffraction patterns.^3^ The difference could be due to the limitations of each technique in modeling dynamic disorder. For example, molecular dynamics can simulate only a small number of molecules compared to that of a real sample. On the other hand, our method does not require any computer-generated molecular structures, but relies on a highly simplified kinematical scattering model to directly quantify disorder from experimental diffraction patterns. Furthermore, some differences in σ values are to be expected due to variations between samples used for the two studies. Nevertheless, the agreement in the order of magnitude for σ suggests that both methods can provide physically meaningful results.

An important assumption in the analysis is treating the terms within the square brackets in eq 4 as being constant. First consider *f*_m_(Δ**k**). The scattering vector magnitude for the diffuse streak, measured with respect to the unscattered beam, is between 0.6 and 1.2 Å^–1^. For carbon, the main constituent element in TIPS pentacene, the atom scattering factor decreases from 1.3 to 0.4 Å within this range. This is a relatively large change in absolute terms but is less of an issue when taking logarithms for a plot of ln(*I*_diff_) versus *k*_∥_^2^. Similarly, the cos(2πΔ**k**·**r**_o_) term in eq 4 is bounded between −1 and 1. This explains the good linear fit observed in panels b and c of Figure 3. For thicker specimens, plasmon scattering is also highly likely, which could result in a “blurring” of the diffraction intensities compared to pure elastic scattering.^27^ However, the characteristic scattering vector magnitude for the (π+σ) plasmon in TIPS pentacene is only 2.2 × 10^–3^ Å^–1^ (see the EELS spectrum in the Supporting Information) and is considerably narrower than the width of diffuse scattering (Figure 3a). Furthermore, the specimen thickness is between 1.3 and 1.5 inelastic mean free paths, indicating that multiple plasmon scattering is also not significant. Therefore, plasmon scattering can be ignored in the present analysis.

A scattering model for static disorder can also be developed along similar lines. The diffuse scattering here is due to imperfect molecular configurations “frozen” into the structure during thin film fabrication. If the atoms in the molecule vibrate independently, the resulting thermal diffuse scattered intensity can overlap with the static diffuse intensity background. However, thermal vibrations in TIPS pentacene are largely correlated,^3,4^ with the entire molecule dynamically oscillating along its long axis direction. This is the linear diffuse streaking analyzed previously and is separate from static disorder. Because the static disorder diffuse scattering is radially symmetric, it is desirable to select virtual apertures in the shape of annular rings. This corresponds to hollow cone illumination in Figure 2a.^20^ To determine the coherence volume, the cos(2πΔ**k**·**r**_*ij*_) term in eq 2 must be integrated over the full range of azimuthal angles ϕ in the diffraction plane, i.e.
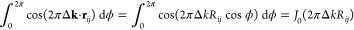
5where *J*_0_ is the zero-order Bessel function of the first kind and *R*_*ij*_ is the magnitude of **r**_*ij*_ in the plane of the specimen. In eq 5, it is reasonably assumed that for high-energy electron diffraction, Δ**k** is predominantly perpendicular to the optic axis. Note that the integration over azimuthal angle ϕ ignores any variation in *f*_m_(Δ**k**), although it will be shown below that this has a negligible effect on the final analysis. The in-plane coherence “volume” for hollow cone illumination is therefore a Bessel function.^20^ Due to static disorder, *R*_*ij*_ will change by a small amount θ from the equilibrium in-plane nearest-neighbor molecule spacing *R*_0_. The Bessel addition theorem gives^26^
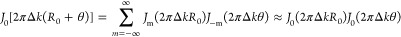
6The final step is because, for small θ values, the Bessel functions are approximately zero unless *m* = 0. From eqs 2, 5, and 6, the mean diffuse intensity *I*_diff_ for a Gaussian distribution of static disorder (standard deviation σ) is given by^26^
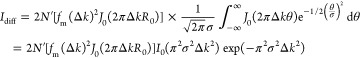
7where *I*_0_ is the zero-order modified Bessel function of the first kind. Figure 4a shows the mean DF intensity as a function of Δ*k* generated from virtual annular apertures of 3 pixels (0.09 Å^–1^) in width. The smallest Δ*k* was limited to 0.27 Å^–1^ to avoid overlap with any strong Bragg reflections. The mean DF intensity was calculated from the same specimen region used for analyzing dynamic disorder. In Figure 4b, the mean DF intensity is divided by 2πΔ*k* to normalize for the virtual annular aperture size. The following function is then least-squares fitted to the data points:
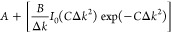
8where the free parameter *A* represents the incoherent background and the expression within the square brackets is the normalized static disorder diffuse intensity (eq 7), with *B* and *C* being fitting parameters. Strictly speaking, *B* is not a constant, because it includes *f*_m_(Δ*k*) and *J*_0_(2πΔ*kR*_0_), although as we shall see, any changes in these variables are suppressed by the dominant exponential term (eq 7). Coefficient *C* is equal to π^2^σ^2^ and yields the static disorder parameter. The data points in Figure 4b are slightly higher than the best fit curve for Δ*k* values between 0.6 and 0.7 Å^–1^, which coincides with the most intense part of the dynamic disorder diffuse streak. Overall, however, the experimental data are well represented by eq 8. The fitted value of σ for static disorder is 1.03 ± 0.03 Å and is significantly larger than the dynamic disorder. It is reasonable for the static disorder to be larger than the dynamic disorder, because intrinsic thermal motion of molecules will “anneal” any defects of similar or smaller magnitude within a relatively short time period. However, a molecular displacement on the order of an angstrom would incur a significant energy penalty and is therefore unexpected. The metastable states created by static disorder can have a multitude of molecular configurations, such as shifting and/or tilting of the pentacene fragments and/or side chains. σ measures the cumulative scattering from all such defects, which may explain its physically unrealistically large value. The presence of large amounts of background diffuse scattering (Figure 1b) does, however, suggest that static disorder in TIPS pentacene cannot be ignored. The results are also fully consistent with experimental observations that processing conditions (and thus the level of disorder) can directly influence the charge carrier mobility.^7^

**Figure 4:**
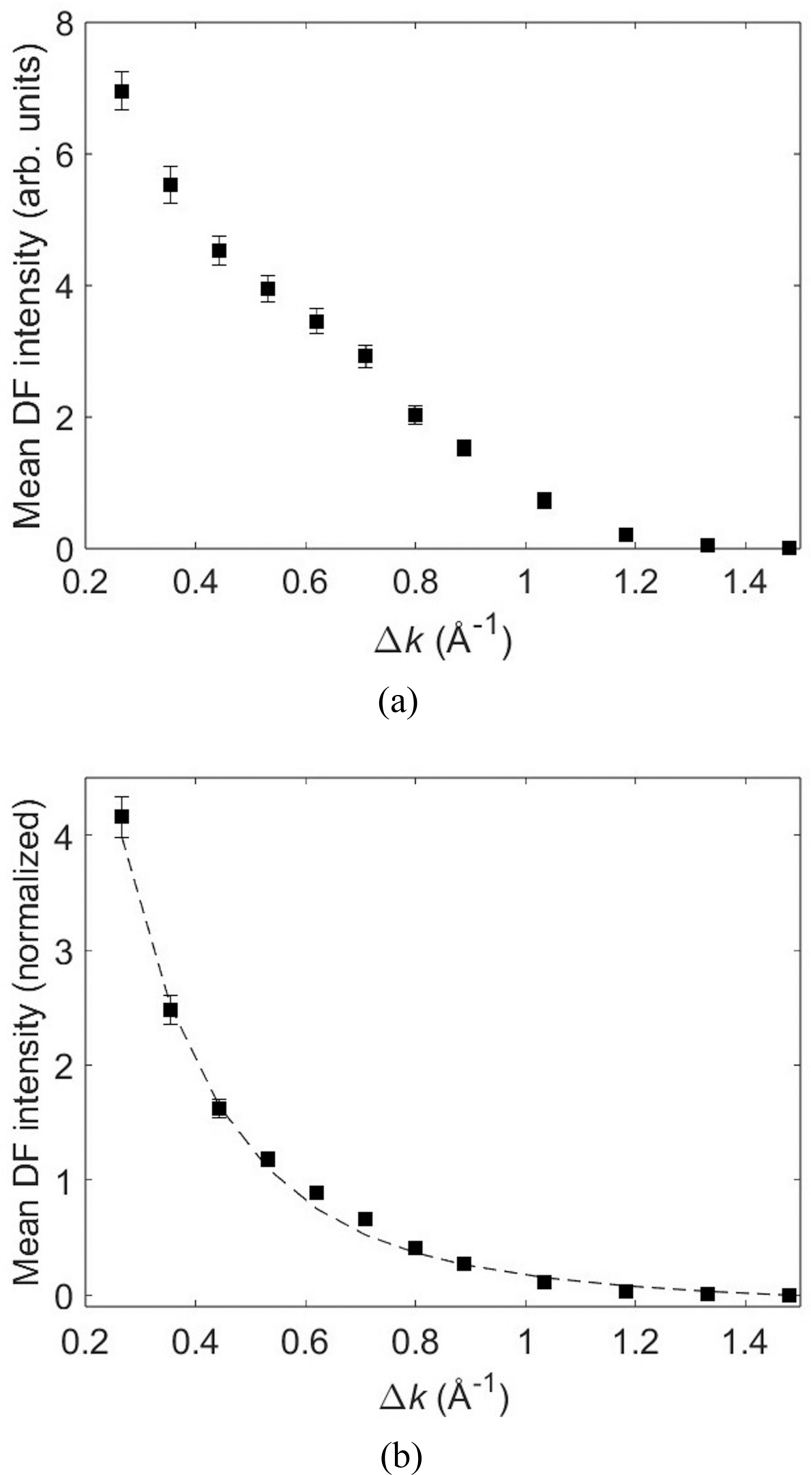
(a) Mean dark-field (DF) intensity plotted as a function of virtual annular aperture radius Δ*k*. In panel b, the mean DF intensity is normalized by dividing by 2πΔ*k*. The dashed line is the best fit curve to the data points using eq 8.

In summary, variable coherence transmission electron microscopy is a promising technique for probing disorder in crystalline molecular solids. In addition to TIPS pentacene, dynamic disorder is present in many other organic materials, such as rubrene,^4^ and is known to significantly alter the charge transfer integrals. Static disorder can also be frozen into the solid during processing and can arise from many different defect configurations. A variable coherence method is developed to quantify the dynamic and static disorder from the diffuse scattering observed in electron diffraction patterns. While the static disorder parameter in our model may not resemble the molecular displacement due to an individual defect configuration and must therefore be treated with caution, we anticipate that this method could nevertheless be used to compare disorder in thin films prepared under different conditions, such as solvents with different boiling points, postdeposition annealing, etc.

## Supporting Information Available

Electron energy loss spectrum for TIPS pentacene (PDF)

## References

[ref1] Noriega R.; Rivnay J.; Vandewal K.; Koch F. P. V.; Stingelin N.; Smith P.; Toney M. F.; Salleo A. A General Relationship Between Disorder, Aggregation and Charge Transport in Conjugated Polymers. Nat. Mater. 2013, 12, 1038–1044. 10.1038/nmat3722. 23913173

[ref2] Fratini S.; Nikolka M.; Salleo A.; Schweicher G.; Sirringhaus H. Charge Transport in High-Mobility Conjugated Polymers and Molecular Semiconductors. Nat. Mater. 2020, 19, 491–502. 10.1038/s41563-020-0647-2. 32296138

[ref3] Eggeman A. S.; Illig S.; Troisi A.; Sirringhaus H.; Midgley P. A. Measurement of Molecular Motion in Organic Semiconductors by Thermal Diffuse Electron Scattering. Nat. Mater. 2013, 12, 1045–1049. 10.1038/nmat3710. 23892786

[ref4] Illig S.; Eggeman A. S.; Troisi A.; Jiang L.; Warwick C.; Nikolka M.; Schweicher G.; Yeates S. G.; Henri Geerts Y.; Anthony J. E.; Sirringhaus H. Reducing Dynamic Disorder in Small-Molecule Organic Semiconductors by Suppressing Large-Amplitude Thermal Motions. Nat. Commun. 2016, 7, 10736 10.1038/ncomms10736. 26898754 PMC4764867

[ref5] Troisi A.; Orlandi G.; Anthony J. E. Electronic Interactions and Thermal Disorder in Molecular Crystals Containing Cofacial Pentacene Units. Chem. Mater. 2005, 17, 5024–5031. 10.1021/cm051150h.

[ref6] de la Perrelle J. M.; Tapping P. C.; Schrefl E.; Stuart A. N.; Huang D. M.; Kee T. W. Singlet Fission Preserves Polarisation Correlation of Excitons. Phys. Chem. Chem. Phys. 2023, 25, 6817–6829. 10.1039/D2CP01943D. 36790866

[ref7] Kim Y.-H.; Lee Y. U.; Han J.-I.; Han S.-M.; Han M.-K. Influence of Solvent on the Film Morphology, Crystallinity and Electrical Characteristics of Triisopropylsilyl Pentacene OTFTs. J. Electrochem. Soc. 2007, 154, H995–H998. 10.1149/1.2783765.

[ref8] Kirkland E. J. Advanced Computing in Electron Microscopy, 2nd ed.; Springer: New York, 2010.

[ref9] Loane R. F.; Xu P.; Silcox J. Thermal Vibrations in Convergent-Beam Electron Diffraction. Acta Cryst. A 1991, 47, 267–278. 10.1107/S0108767391000375.

[ref10] Treacy M. M. J.; Gibson J. M. Variable Coherence Microscopy: A Rich Source of Structural Information from Disordered Materials. Acta Cryst. A 1996, 52, 212–220. 10.1107/S0108767395012876.

[ref11] Voyles P. M.; Gibson J. M.; Treacy M. M. J. Fluctuation Microscopy: A Probe of Atomic Correlations in Disordered Materials. J. Electron Microsc. 2000, 49, 259–266. 10.1093/oxfordjournals.jmicro.a023805. 11108048

[ref12] Gibson J. M.; Treacy M. M. J.; Voyles P. M. Atom Pair Persistence in Disordered Materials from Fluctuation Microscopy. Ultramicroscopy 2000, 83, 169–178. 10.1016/S0304-3991(00)00013-9. 10841332

[ref13] Voyles P. M.; Abelson J. R. Medium-Range Order in Amorphous Silicon Measured by Fluctuation Electron Microscopy. Sol. Energy Mater. Sol. Cells 2003, 78, 85–113. 10.1016/S0927-0248(02)00434-8.

[ref14] Zhang P.; Maldonis J. J.; Besser M. F.; Kramer M. J.; Voyles P. M. Medium-Range Structure and Glass Forming Ability in Zr-Cu-Al Bulk Metallic Glasses. Acta Mater. 2016, 109, 103–114. 10.1016/j.actamat.2016.02.006.

[ref15] Hilke S.; Rösner H.; Geissler D.; Gebert A.; Peterlechner M.; Wilde G. The Influence of Deformation on the Medium-Range Order of a Zr-based Bulk Metallic Glass Characterised by Variable Resolution Fluctuation Electron Microscopy. Acta Mater. 2019, 171, 275–281. 10.1016/j.actamat.2019.04.023.

[ref16] Zhao G.; Buseck P. R.; Rougée A.; Treacy M. M. J. Medium-Range Order in Molecular Materials: Fluctuation Electron Microscopy for Detecting Fullerenes in Disordered Carbons. Ultramicroscopy 2009, 109, 177–188. 10.1016/j.ultramic.2008.10.006. 19062186

[ref17] Faure P.; Micu A.; Perahia D.; Doucet J.; Smith J. C.; Benoit J. P. Correlated Intramolecular Motions and Diffuse X-Ray Scattering in Lysozyme. Nat. Struct. Mol. 1994, 1, 124–128. 10.1038/nsb0294-124. 7656016

[ref18] Ayyer K.; Yefanov O. M.; Oberthür D.; Roy-Chowdhury S.; Galli L.; Mariani V.; Basu S.; Coe J.; Conrad C. E.; Fromme R.; Schaffer A.; Dörner K.; James D.; Kupitz C.; Metz M.; Nelson G.; Xavier P. L.; Beyerlein K. R.; Schmidt M.; Sarrou I.; Spence J. C. H.; Weierstall U.; White T. A.; Yang J.-H.; Zhao Y.; Liang M.; Aquila A.; Hunter M. S.; Robinson J. S.; Koglin J. E.; Boutet S.; Fromme P.; Barty A.; Chapman H. N. Macromolecular Diffractive Imaging Using Imperfect Crystals. Nature 2016, 530, 202–206. 10.1038/nature16949. 26863980 PMC4839592

[ref19] Meisburger S. P.; Case D. A.; Ando N. Diffuse X-Ray Scattering From Correlated Motions in a Protein Crystal. Nat. Commun. 2020, 11, 1271 10.1038/s41467-020-14933-6. 32152274 PMC7062842

[ref20] Treacy M. M. J.; Gibson J. M. Coherence and Multiple Scattering in “Z-contrast” Images. Ultramicroscopy 1993, 52, 31–53. 10.1016/0304-3991(93)90020-X.

[ref21] Ophus C. Four-Dimensional Scanning Transmission Electron Microscopy (4D-STEM): From Scanning Nanodiffraction to Ptychography and Beyond. Microsc. Microanal. 2019, 25, 563–582. 10.1017/S1431927619000497. 31084643

[ref22] Panova O.; Ophus C.; Takacs C. J.; Bustillo K. C.; Balhorn L.; Salleo A.; Balsara N.; Minor A. M. Diffraction Imaging of Nanocrystalline Structures in Organic Semiconductor Molecular Thin Films. Nat. Mater. 2019, 18, 860–865. 10.1038/s41563-019-0387-3. 31160799

[ref23] Mu X.; Mazilkin A.; Sprau C.; Colsmann A.; Kübel C. Mapping Structure and Morphology of Amorphous Organic Thin Films by 4D-STEM Pair Distribution Function Analysis. Microscopy 2019, 68, 301–309. 10.1093/jmicro/dfz015. 31220309

[ref24] Yi F.; Voyles P. M. Effect of Sample Thickness, Energy Filtering, and Probe Coherence on Fluctuation Electron Microscopy Experiments. Ultramicroscopy 2011, 111, 1375–1380. 10.1016/j.ultramic.2011.05.004. 21864780

[ref25] Egerton R. F. Electron Energy-Loss Spectroscopy in the Electron Microscope; Plenum Press: New York, 1996.

[ref26] Gradshteyn I. S.; Ryzhik I. M. Tables of Integrals, Series and Products; Academic Press: New York, 1980.

[ref27] Mendis B. G. An Inelastic Multislice Simulation Method Incorporating Plasmon Energy Losses. Ultramicroscopy 2019, 206, 112816 10.1016/j.ultramic.2019.112816. 31377522

[ref28] Momma K.; Izumi F. VESTA: A Three-Dimensional Visualisation System for Electronic and Structural Analysis. J. Appl. Crystallogr. 2008, 41, 653–658. 10.1107/S0021889808012016.

