# Supplementary material for: Quantifying Molecular Disorder in Tri-Isopropyl Silane (TIPS) Pentacene Using Variable Coherence Transmission Electron Microscopy
Source: J Phys Chem Lett. 2023 Sep 6;14(36):8183–90. doi: 10.1021/acs.jpclett.3c01344 (PMC10510430; doi:10.1021/acs.jpclett.3c01344)
Supplement: Supplementary file 1 — jz3c01344_si_001.pdf [file jz3c01344_si_001.pdf]

Quantifying Molecular Disorder in Tri-Isopropyl Silane (TIPS) Pentacene using Variable Coherence Transmission Electron Microscopy

F Alanazi[a], AS Eggeman[b] , K Stavrou[a], A Danos[a], AP Monkman[a], BG Mendis[a*]

a. *Dept. Of Physics, Durham University, South Road, Durham, DH1 3LE, UK.*
b. *Dept. of Materials, University of Manchester, Oxford Road, M13 9PL, UK.*

* Corresponding author (b.g.mendis@durham.ac.uk)

Supporting Information

(1) Electron energy loss spectrum for TIPS pentacene.

The electron energy loss spectrum (EELS) for drop cast TIPS pentacene is shown below. The feature at ~22 eV is the (π+σ) bulk plasmon. The plasmon characteristic scattering angle is therefore 5.5 x 10^{-2} mrad, or equivalently 2.2 x 10^{-3} $Å^{-1}$ scattering vector magnitude for 200 kV incident electrons.

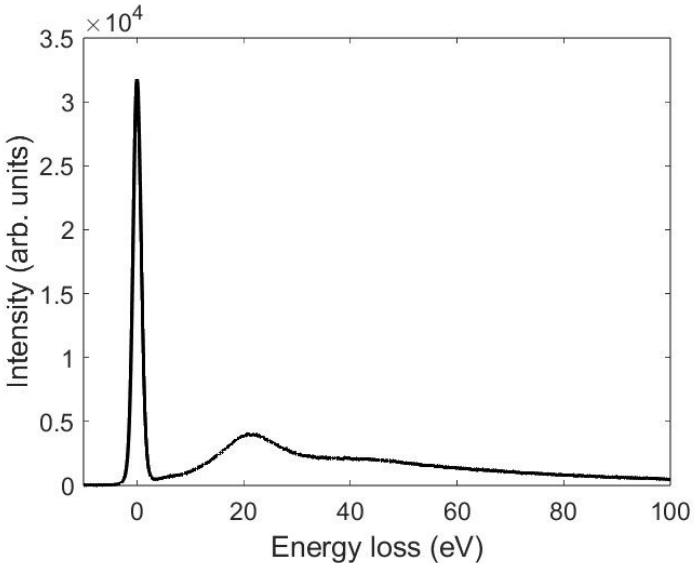

Figure S1: EELS spectrum for TIPS pentacene.
